# Application of a Body Shape Index as an Anthropometric Predictor of Cardiometabolic Risks in Children and Adolescents (Systematic Review)

**DOI:** 10.17691/stm2024.16.5.06

**Published:** 2024-10-30

**Authors:** S.A. Todorova

**Affiliations:** MD, PhD, Department of Internal Diseases and General Medicine, Faculty of Medicine Trakia University, 11 Armeiska St., Stara Zagora, 6000, Bulgaria

**Keywords:** a body shape index, children and adolescents, obesity, cardiometabolic risks

## Abstract

A body shape index (ABSI) is a novel anthropometric measure associated with body obesity. However, the research on the relationship between the new anthropometric risk index and metabolic disorders and cardiovascular alterations in the pediatric population is limited.

**The aim of the study** was to perform a systematic review and meta-analysis of the associations between cardiometabolic risk and ABSI and evaluate its discrimination capacity.

**Materials and Methods:**

A systematic search and analysis of the available scientific literature were conducted to identify relevant articles published up to March 2024 in Web of Science, Science Direct, Scopus, ResearchGate, and PubMed. All full-text publications reporting an association between cardiometabolic risk and ABSI in children and adolescents were considered eligible and reviewed in detail. The reported results and statements were summarized using meta-analysis methods.

**Results:**

A systematic review of 17 cross-sectional studies involving 31,849 children and adolescents, predominantly from Asian countries, was conducted. Nine studies included fewer than 1,000 subjects, raising questions about the representativeness of the samples and the reliability of the reported results. The data regarding the relationship between ABSI and cardiometabolic risk remain controversial. Even when applying age- and sex-adjusted ABSI, and when compared with other established or newly introduced anthropometric measurements, the discriminatory capacity of ABSI remains uncertain.

**Conclusions:**

The findings to date highlight the variable discriminating capacity of ABSI as a predictor of cardiometabolic risk in children and adolescents.

## Introduction

For decades in a row, the body mass index (BMI) calculated from body weight and height and waist circumference (WC) indicating abdominal fat accumulation are standard anthropometric measures of obesity and metabolic syndrome (MetS) among children and adolescents in the recommendations of World Health Organization (WHO) and International Diabetic Federation (IDF). BMI mostly refects total body fat rather than the regional distribution of fat, which is associated with elevated health risk [1-3]. In young healthy individuals, the indicated index presents an inadequate assessment, measuring not the real fat mass, but skeletal muscle mass [4-7]. To date, several country-specific pediatric WC percentage cutoffs have been developed, and WC cutoffs for defining central obesity in children and adolescents are in the process of being developed and proposed based on studies involving multiple populations from different countries and regions [8-13]. Additionally, there are other indicators for evaluating the risk of pediatric obesity and related cardiovascular risks that are useful for application, but with still controversial evidence of their precision and verifiability. A body shape index (ABSI) is a novel introduced metric for assessing obesity among children and adolescents, gaining increasing popularity and usability in foreign and national scientific studies [14–16].

**The aim of the study** was to perform a systematic review and meta-analysis of the associations between cardiometabolic risk and ABSI and evaluate its discrimination capacity.

## Materials and Methods

### Study design

A systematic review was carried out by searching online databases from various credible scientific sources of verified and reliable information.

### Study settings

The time frame of search, selection, and analysis was from January 1 to March 1, 2024.

### Inclusion criteria

The following criteria were applied in the selection of the searched appropriate articles:

Only children and adolescents with or without overweight/obesity were included;

Calculation of ABSI as an anthropometric indicator for predicting abdominal obesity, dyslipidemia, MetS, arterial hypertension, and cardiovascular alterations;

The comparison of ABSI with other new and/or established anthropometric indices regarding their discriminatory capacity to predict cardiometabolic risks in children and adolescents.

### Exclusion criteria

The study removed all research on individuals over 18 years old and on children and adolescents affected by genetic or syndromic obesity. The studies with lack of ABSI as an anthropometric index applied to children dropped out of the selection. All non-original studies, case series, conference abstracts, and unavailable full texts were also excluded.

### Concept of searching, sampling methods, and data analysis technique

A systematic review was performed in globally-recognized databases Web of Science, Science Direct, Scopus, Research Gate, PubMed and available cross-references over an eleven-year time period (2012–2023). The procedural steps of this study were based on established guidelines on the preferred reporting items for systematic reviews and metaanalyses (PRISMA) [17]. The combinations of the following terms and sentences were used when discovering and identifying relevant publications:

1. ABSI and pediatric obesity;
2. ABSI and obesity in children and adolescents;
3. ABSI and MetS in children and adolescents;
4. ABSI and arterial hypertension in children and adolescents;
5. ABSI and pediatric hypertension;
6. ABSI and elevated blood pressure in children and adolescents.

Each article title and/or abstract of the related articles in the literature databases was screened to assess its potential relevance. All found duplicates were excluded. Selected full-text articles were downloaded for further detailed review, analysis of results, and determination of eligibility.

## Results

A total of 520 records was obtained from 5 above-mentioned literature databases and cross-referencing. The details of the selection of scientific articles based on the inclusion criteria are presented in Figure.

**Figure:**
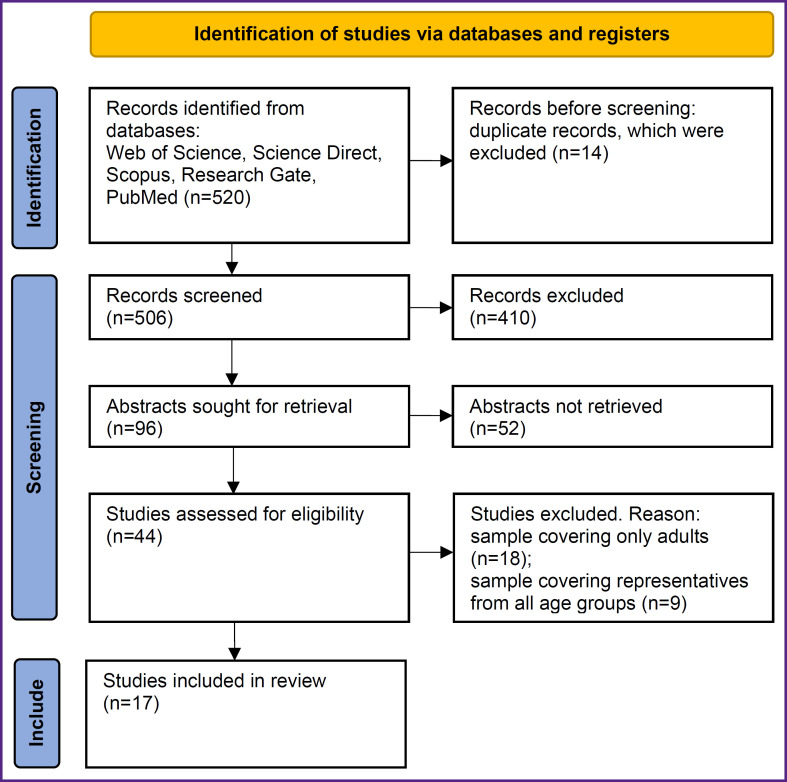
Flow diagram of the steps of the systematic review according to the guidelines of PRISMA

All examined research and offcially published national studies that have been analyzed are legitimate and similar in nature. Fourteen duplicates were excluded. 506 titles of articles and abstracts were screened for potential eligibility, among which 410 were deleted as irrelevant and mismatched records with our set indications for inclusion. 44 full-text articles were reviewed. 27 publications were further excluded because their samples included only adults or representative of all age groups. 31,849 individuals from 17 cross-sectional studies were included in this meta-analysis. Table shows additional information on the involved articles [14–16, 18–31]. The analyzed surveys were published from 2013 to 2022. The number of participants in the samples varied from 197 to 14,008. The age of individuals ranged from 3.0 to 19.9 years. Nine articles had subjects less than 1000. Ten surveys were conducted in Asian countries with a predominant number of respondents (China, India, Iran, Malaysia, Korea). The remaining cross-sectional studies were carried out in Italy, Spain, Portugal, and Brazil. All articles contain data from conducted studies comparing the discriminatory capacity of ABSI with other newly developed or established anthropometric indicators of obesity applied as predictors of cardiometabolic risk in children and adolescents.

**Table:** Characteristics of the included studies

Reference	Study design	Sample size (n)	Age (years old)	Country	Assessment of the association with metabolic risk compared to other indices*	Assessment of the association with cardiovascular risk compared to other indices*
Perona et al., 2019 [14]	Cross-sectional study	981	11–16	Spain	< sensitivity	—
Yazdi et al., 2020 [15]	Cross-sectional study (CASPIAN-V nationwide study)	14,008	7–18	Iran	—	< sensitivity
Ge et al., 2022 [16]	Cross-sectional study + meta-analysis	3150	7–17	China	—	<sensitivity
Vizzuso et al., 2021 [18]	Observational cross-sectional study	637	8–15	Italy	< sensitivity	—
Cristine Silva et al., 2020 [19]	Cross-sectional study	1069	12–17	Brazil	≥ sensitivity (ABSI, adjusted ABSI)	< sensitivity (ABSI, adjusted ABSI)
Giudici et al., 2017 [20]	Cross-sectional study	197	14–18	Brazil	≤ sensitivity	—
Mameli et al., 2018 [21]	Observational cross-sectional study	217	2–18	Italy	≥ sensitivity (ABSI z score)	≥ sensitivity (ABSI z score)
Leone et al., 2020 [22]	Cross-sectional study	403	7–19.9	Italy	≥ sensitivity (ABSI z score)	—
Kasaeian et al., 2021 [23]	Cross-sectional study	4200	7–18	Iran	> sensitivity	> sensitivity
Duncan et al., 2013 [24]	Cross-sectional study	445	10–17	Portugal	—	≥ sensitivity
Talwar et al., 2020 [25]	Cross-sectional study	215	11–18	India	—	≥ sensitivity
Tong and Han, 2019 [26]	Cross-sectional study	1387	7–17	China	—	< sensitivity (ABSI-(C))
Te e et al., 2020 [27]	Cross-sectional study	513	12–16	Malaysia	—	< sensitivity
Xu et al., 2015 [28]	Cross-sectional study	562	10–17	China	≥ sensitivity	< sensitivity
Chen et al., 2022 [29]	Cross-sectional study	1587	3–17	China	< sensitivity	< sensitivity
Lek et al., 2016 [30]	Cross-sectional study	1755	10–17	China	< sensitivity	< sensitivity
Kwak et al., 2021 [31]	Cross-sectional study (KNHANES)	1023	10–19	Korea	< sensitivity	< sensitivity

N o t e:* the assessment is based on the conducted statistical analyzes presented in the research ABSI z score, adjusted ABSI, and ABSI-(C) are modified ABSI according to gender and/or age applied in the study.

## Discussion

A body shape index is a new and improved index proposed in 2012 by N.Y. Krakauer and J.C. Krakauer [32]. It was developed as a method to quantitatively assess the risk associated with abdominal obesity (the ratio of WC to height and BMI), based on the authors’ analysis of study results (patient history, medical examination, mortality rates) obtained during 1999– 2004 by the United States National Health and Nutrition Examination Survey (NHANES). Initially, in the USA population as sampled in NHANES, ABSI predicted mortality risk across age, sex, and weight. Krakauer and co-author [32] shared that the derived metric can be used as a predictor of many aspects of health, especially abdominal obesity, MetS, and cardiovascular conditions. ABSI formula was defined as the ratio of WC to BMI^2/3^ **·** height^1^/^2^. It is an easy and informative risk metric and more appropriate than BMI for a given condition or population. The ABSI obtained from the adult population is difficult to apply to the children and adolescents. The annual height, weight, and BMI velocity in the pediatric population change with age and fat distribution in adolescents varies with age, sex, pubertal development, and nutritional status [33]. Furthermore, visceral fat, due to its location, hormonal activity, and metabolic characteristics, contributes to impaired metabolism in children and adolescents. With central body fat deposition, the probability of having cardio-vascular risk factors also increases. Namely, the corrected for adolescents ABSI, which validates the ratio of abdominal to peripheral fat, was specifically developed to highlight the importance of WC in abdominal obesity predisposing to metabolic and cardiovascular changes. To date, relatively few studies, with both small and large sample sizes, have demonstrated a varying (high or low, positive or negative) correlation of ABSI with MetS, abdominal obesity, and cardiovascular risks among children and adolescents.

Perona and co-authors [14] opined that ABSI should not be used as a predictive index for MetS in adolescents of both genders, citing its reported low ability to discriminate subjects with this condition in their research performed on 981 Spanish teenagers (11-16 years old). The parameters and anthropometric indexes WC, BMI, weight-to-hip ratio (WHR), and waist-to-height ratio (WHtR) with the exception of ABSI analyzed in this cross-sectional study showed higher values among the adolescents with MetS. Research conducted in Italy concluded that ABSI together with BMI z-score and WHR did not differ in 637 obese Caucasian children and adolescents aged 8 to 15 years with or without diagnosed MetS [18]. The researchers share another metric, visceral adiposity index (VAI), as a promising tool to identify MetS in children and adolescents with obesity when compared to models including sex, age, and BMI z-score, ABSI or WHR. The following American survey among 1069 Brazilian participants aged 12–17 years explored the capacity of seven anthropometric indices (BMI, WC, WHR, conicity index (CoI), ABSI, adjusted ABSI for adolescents (adjusted ABSI), and body roundness index (BRI)) in predicting individual cardiovascular and metabolic risk [19]. For MetS, all the indices showed very good to excellent reliability, but limited accuracy for cardiovascular risk markers. Cristine Silva et al. [19] concluded that precision of CoI, BSI, adjusted BSI, and BRI is not superior to BMI, WC, and WHR in predicting cardiometabolic risks. The corrected for adolescents ABSI (ABSI-adolescents), but not ABSI, were related to the adiponectin-to-leptin ratio and to markers of glucose metabolism, but not more strongly than BMI and WC in other Latin research including 197 Brazilian adolescents aged 14–18 years [20]. In the cited study, BMI showed the strongest association with the exanimated indicators — insulin, insulin resistance (HOMA-IR), pancreatic β-cell function (HOMA-β%), and the quantitative insulin sensitivity check index (QUICKI).

On the other hand, observational cross-sectional study among 217 overweight and obese children and adolescents aged 2–18 years showed significant correlation of ABSI z score with 10 out of 15 cardiometabolic risk markers like fasting insulin, insulin resistance (HOMA-IR), pancreatic β-cell function (HOMA-β%), total cholesterol, LDL cholesterol, triglycerides (TG), alanine aminotransferase, triglycerides/ HDL cholesterol ratio (TG/HDL) [21]. The reported results demonstrated ABSI values, which were converted to age-and sex-specific z scores, were positively associated with glucose metabolism indexes and the whole lipid profile along with a biomarker of atherogenic dyslipidemia and altered cardiometabolic risk — TG/HDL. Leone et al. [22] observed that the inclusion of ABSIz improved the prediction of MetS compared to BMIz alone in Italian children and adolescents. Due to the small sample size (403 Caucasian children and adolescents aged 7–19.9 years) in a cross-sectional study, the authors reported an inability to identify specific cut-off for ABSI to define the obesity, central obesity, and consequently the cardiometabolic risk. In nationwide research on 4200 Iranian students who were 7–18 years old, Kasaeian et al. [23] found a statistically significant of ABSI with overweight, generalized, and abdominal obesity. Even more, ABSI has sufficient discriminating capacity to be applied as an anthropometric indicator for determining the risk of MetS and arterial hypertension noted by researchers.

The results from a study of 445 Portuguese adolescents aged 10 to 17 years have ascertained better capabilities of ABSI than WC and BMI in predicting the risk of arterial hypertension [24]. The authors of study noted that ABSI predicted a larger extent of the variance in both systolic and diastolic blood pressure for boys and girls. As early as 2013, the explorers suggested that when examining the effect of weight status on arterial blood pressure, it should consider using ABSI together with BMI. Despite the small size and selection-specific sample of 215 Indian boys (11–18 years old) in the survey of Talwar et al. [25], regression analysis depicted the systolic blood pressure of subjects was associated significantly with BMI followed by WC, ABSI, and a body adiposity index (BAI), and diastolic blood pressure revealed highest correlation with BMI, ABSI, and WC. The authors reported BMI predicted more variances in both systolic blood pressure and diastolic blood pressure.

Most of the recent studies on adolescents failed to find a significant association of ABSI with arterial blood pressure. Some of them continue to confirm the stronger relationship of BMI with cardiometabolic risk factors compared to the newly introduced ABSI. A large nationwide study involving 14,008 Iranian children in the age range of 7–18 years reported WC, WHR, or ABSI in combination with BMI did not improve predictive power to identify subjects at higher risk of elevated arterial blood pressure [15]. Of the four anthropometric indices, with exception of ABSI, BMI, WC, and WHR showed a nearly similar predictive power for identifying arterial hypertension. Data from another Asian survey of 1387 children aged 7–17 years again showed a low level of accuracy of the two new indices, ABSI and BAI, in predicting high blood pressure [26]. Even after appropriate redefinition of the ABSI for Chinese children and adolescents (ABSI-(C)), it has demonstrated less precision in estimating hypertension and pre-hypertension. Referring to the results, Tong and Han [26] shared that for Chinese children and adolescents, BMI remains an important adiposity index for epidemiological studies. ABSI presented the worst predictive power and sensitivity in identifying high blood pressure among 513 Malaysian adolescents (aged 12–16 years) in the study of Tee et al. [27]. Considering both sensitivity and specificity, WHtR was the most accurate indicator to predict the presence of arterial hypertension alongside BMI among male and female adolescents. The reasons for the underperformance of ABSI in predicting chronic diseases, especially arterial hypertension, are most likely to be found in the variations in age, gender, and ethnicity determining the use of locally adapted cut-off values for assessment.

Xu et al. [28] also expressed an opinion in favor of BMI, referring to the results of their research among 562 individuals aged 10–17 years. If the assessment is initially motivated by a concern for high blood pressure and pre-hypertension, BMI is sufficient. On the other hand, the ABSI-adolescents and the WHtR were more associated with glycated hemoglobin and pre-diabetes than BMI. The explorers proposed the ABSI-adolescents formula, applying specific scaling exponents to standardized WC for BMI and height among Chinese participants. When calculating five anthropometric indicators (BMI, WC, BRI, WHtR, and ABSI) in 1,587 Chinese participants aged 3–17 years, Chen et al. [29] came to the conclusion that in the 7–17 years-old group, WHtR and BRI were significantly better in identifying hypertension, dyslipidemia, abdominal obesity in both genders compared with BMI, ABSI, and ABSI-adolescents, which has been updated on the scaling exponents in ABSI by Xu et al. [28]. And overall, ABSI showed the worst ability in distinguishing all the single or clustered cardiometabolic risk factors. A study among 1755 teenagers from three ethnic groups also examined the applicability of ABSI-adolescents and its association with cardiometabolic outcomes compared to WC, BMI, WHR, and WHtR [30]. It observed variety of body proportionality and WC-BMI-height profles between the ethnic contingents different from the previously proposed by Xu et al. [28] ABSI-adolescents. The results showed WC and WC-based indices were associated with blood pressure and fasting blood glucose in adolescents of three ethnicities. Based on the results of a large-scale study among 3150 Chinese participants aged 7–17 years, Asian researchers shared the view that in predicting pediatric arterial hypertension, original ABSI and modifed ABSI do not perform as well as traditional anthropometric indicators, such as BMI and WC [16]. The mentioned cross-sectional study was combined with a brief meta-analysis comparing the predictive power for high blood pressure of BMI, WC, original ABSI, and modifed ABSI. In this way, the cited authors proved their hypothesis that BMI remains the optimal indicator in pediatric screening of arterial hypertension. Further studies are required to verify the current outcomes’ accuracy and whether the results are matched by race and sex. ABSI is difficult to apply to the pediatric contingent because annual height, weight, and BMI rates change with age. Additionally, fat distribution in adolescents varies with age, sex, puberty, genetic and environmental influences, diet patterns, and physical activity. Kwak et al. [31] proved the variability according to age and different change patterns between boys and girls of the mean ABSI was associated with growth and pubertal development. The results of the conducted Korea National Health and Nutrition Examination Survey covering 1023 participants aged 10– 19 years concluded that it is appropriate to use the BSI obtained using different scaling indicators depending on age and gender in teenagers.

Research from different nationalities had variations in the conclusions regarding the superiority of one or the other anthropometric indices and the related cut-off values to determine abdominal obesity and the related MetS and cardiovascular alterations. The contradiction and discrepancy in reviewed studies are likely the result of diversity in the research design, characteristics of the sample, and applied methodologies. There are too few studies worldwide comparing ABSI with other indices used for decades in connection to cardiometabolic risk among children and adolescents. There is also a lack of research investigating and analyzing the relationship of ABSI with arterial blood pressure values and levels of substances refecting glucose metabolism and lipid profile. All analyzed articles presented results from studies conducted in a given geographic region or country. In most of the studies, the sample included a small number of participants making them unrepresentative and unsure of the claims that were reported. The authors summarize that a series of determinants such as geographical area and ethnicity, age and gender, puberty and hormonal status, genes and heredity, lifestyle, and socio-economic status of the participants can affect the results of each study considered. For future studies, the confirmation or refutation of these findings will require further investigation using larger sample by including children and adolescents from different countries and regions. More scientific data need to be collected to establish ABSI cut-off values that are universally applicable and suitable for correct interpretation and diagnosis of central obesity and its associated cardiovascular and metabolic changes in children and adolescents.

### Limitations

Limitations need to be shared are primarily the small number of studies conducted to date regarding the discriminatory capacity of ABSI as a novel predictor of cardiometabolic risk in children and adolescents. A significant part of the results presented are based on studies covering individuals from Asian countries. Too few data on the precision of anthropometric measure have been reported in other countries and regions, which determines the need for more national and international studies and subsequent meta-analyses.

## Conclusion

ABSI, which expresses waist circumference relative to height and weight, has been proposed as a novel and more accurate method to quantify the specific risk associated with central obesity and related to it cardiometabolic risk among adolescents. The findings from the research conducted to date shed light on the varying discriminative capacity of the ABSI. A study with a representative sample including broader populations from different countries and geographical regions is needed to investigate in depth the predictive capacity of the metric under consideration.
